# Intramuscular spindle cell lipoma of the deltoid: a case report

**DOI:** 10.1186/s13256-014-0509-0

**Published:** 2015-02-19

**Authors:** Naoki Mizoshiri, Toshiharu Shirai, Ryu Terauchi, Yuji Arai, Hiroyoshi Fujiwara, Eiichi Konishi, Hiroyuki Tsuchiya, Toshikazu Kubo

**Affiliations:** 1 Department of Orthopaedics, Graduate School of Medical Science, Kyoto Prefectural University of Medicine, Kamigyo-ku, Kyoto 602-8566 Japan; 2 Department of Orthopaedic Surgery, Graduate School of Medical Science, Kanazawa University, 13-1 Takaramachi, Kanazawa, 920-8641 Japan; 3 Department of Pathology, Kyoto Prefectural University of Medicine, Kamigyo-ku, Kyoto 602-8566 Japan

**Keywords:** Deltoid, Intramuscular spindle cell lipoma, Soft tissue tumor

## Abstract

**Introduction:**

Spindle cell lipoma is an uncommon adipocytic tumor. Intramuscular lesions of this tumor are very rare. In this report, we describe a case of a patient with intramuscular spindle cell lipoma localized in a deltoid.

**Case presentation:**

A 58-year-old Japanese man visited us because of a soft tissue mass on the lateral aspect of the left shoulder that had been noticed 2 years prior. The spherical tumor, which measured 5cm×4cm, was elastic and firm on palpation and immobile. Magnetic resonance imaging revealed that the lesion was localized in the left deltoid muscle. A needle biopsy was performed to make a histological diagnosis. With a pre-operative diagnosis of intramuscular lipoma, we removed the tumor with the patient under general anesthesia. The tumor was removed with surrounding musculature and fascia. The pathological diagnosis was intramuscular spindle cell lipoma in the left deltoid muscle.

**Conclusions:**

There are several kinds of lipomas. Spindle cell lipoma is a relatively rare variant (1.5% of all adipocytic neoplasms) that is histologically distinct and characterized by the replacement of mature fat by a mixture of mature adipocytes and undifferentiated spindle cells. There are only five other reported cases of intramuscular spindle cell lipoma in the literature, to our knowledge. The case of our patient is very interesting, as to date there have been few reported patients with a diagnosis of an intramuscular spindle cell lipoma in a deltoid.

**Electronic supplementary material:**

The online version of this article (doi:10.1186/s13256-014-0509-0) contains supplementary material, which is available to authorized users.

## Introduction

Spindle cell lipoma (SCL) is an uncommon adipocytic tumor that was first described by Enzinger and Harvey in 1975 [1]. It occurs predominantly in men between 45 and 70 years of age and in most cases is found in the subcutaneous tissue of the neck, shoulder or back [1-3]. SCLs are fairly well-circumscribed, subcutaneous tumors that are composed of varying proportions of mature fat cells, small and uniform spindle cells and eosinophilic collagen bundles [1,3,4]. The stroma can range from collagenous to myxoid [3,4]. Intramuscular lesions of this tumor are very rare. In this report, we present a case of a patient with intramuscular SCL localized in the left deltoid muscle.

## Case presentation

**Figure 1:**
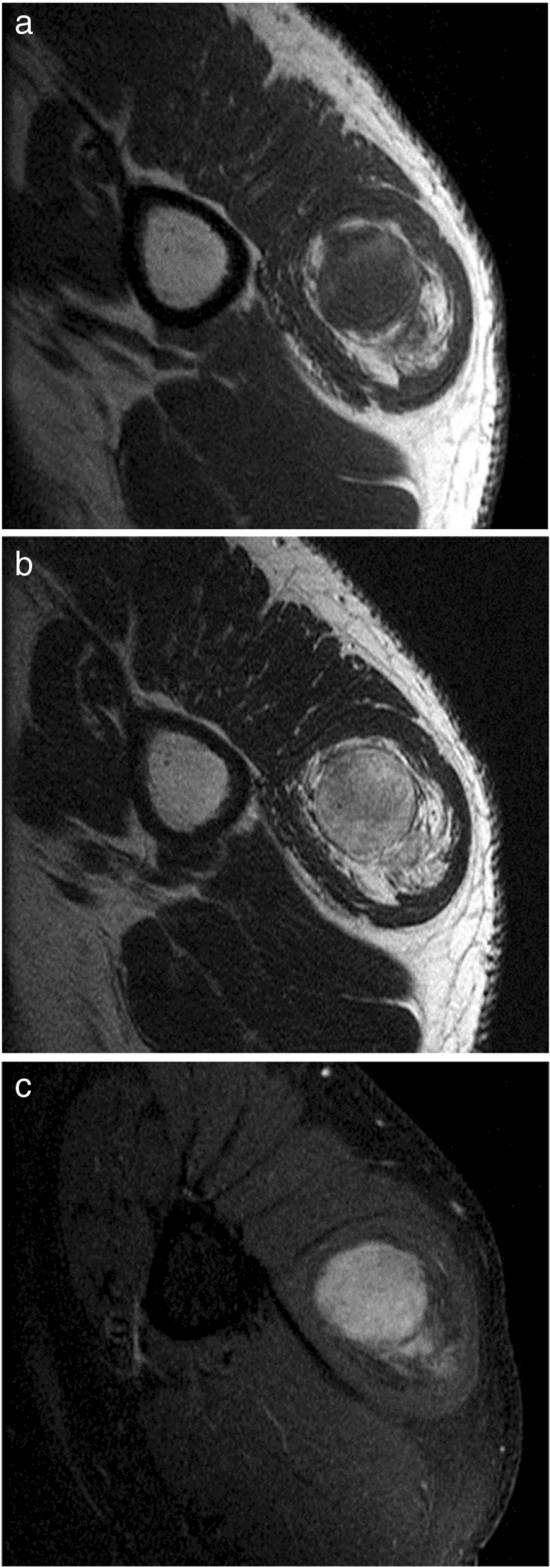
**Signal intensity of the tumor on magnetic resonance imaging scans. (a)** On this T1-weighted image, the tumor shows low signal intensity or isointensity to skeletal muscle at the center and high signal intensity at the periphery of the lesion. **(b)** On this T2-weighted image, the tumor shows high signal intensity. **(c)** On this gadolinium-enhanced image, the tumor shows high signal intensity.

A 58-year-old Japanese man visited us because of a soft tissue mass on the lateral aspect of the left shoulder that had been noticed 2 years prior. His past and family histories were not contributory. All other laboratory examinations showed no abnormalities. The spherical tumor, measuring 5cm×4cm, was elastic and firm on palpation and immobile. There were no other findings of inflammation. Magnetic resonance imaging (MRI) revealed that the lesion was localized in the left deltoid muscle. On MRI scans, the tumor showed low signal intensity or isointensity to skeletal muscle at the center. It showed high signal intensity at the periphery of the lesion on a T1-weighted image (Figure 1a). It also showed high signal intensity on a T2-weighted image (Figure 1b) and a gadolinium-enhanced image (Figure 1c). However, the signal intensity was substantially lower than that of normal subcutaneous adipose tissue. On the basis of these radiological findings, we suspected a lipogenic tumor. A needle biopsy was performed to make a histological diagnosis. Histologically, the tumor was a lipomatous tumor consisted mostly of atypical lipocytes with a slightly greater variation in size and shape than those of normal fat. Although the atypism of the cells was slight and mitoses were not seen, a few lipoblast-like cells and fibroblast-like cells were seen. The results of MDM2 immunostaining were negative.

**Figure 2:**
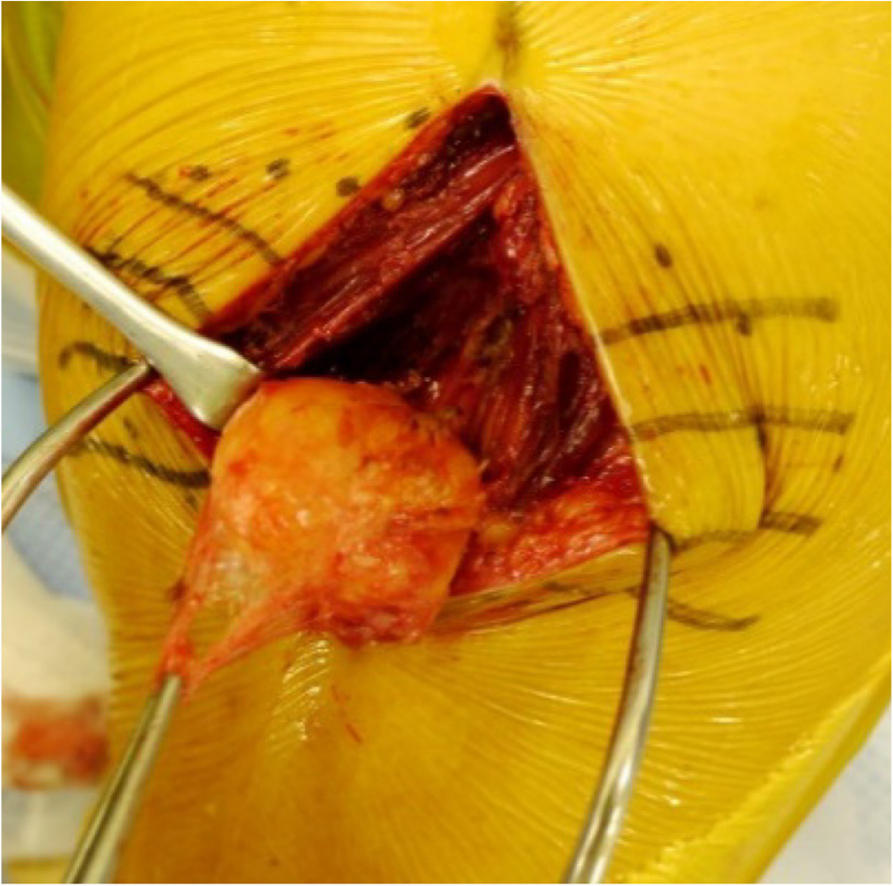
**Intra-operative photograph of the tumor.** During surgery, it was confirmed that the whole lesion was localized underneath the fascia and embedded within the deltoid.

**Figure 3:**
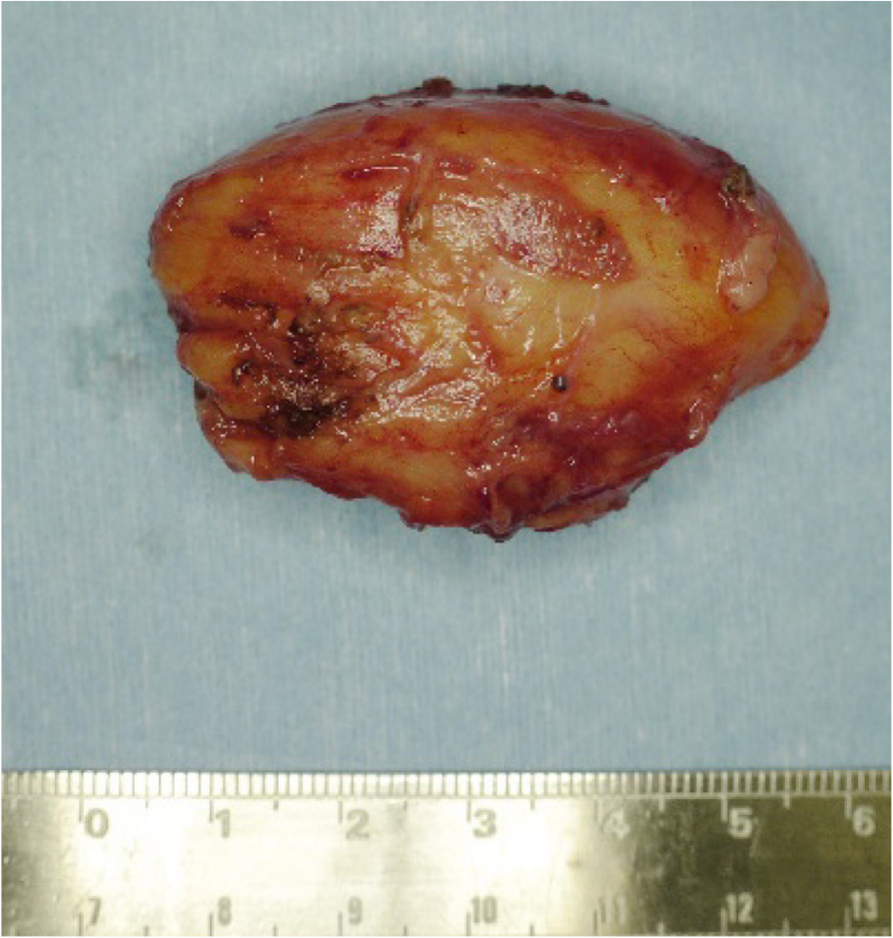
**Post-operative photograph of the excised tumor.** The excised tumor was 5cm×4cm×3cm in size and had a yellowish color like that of a lipoma.

**Figure 4:**
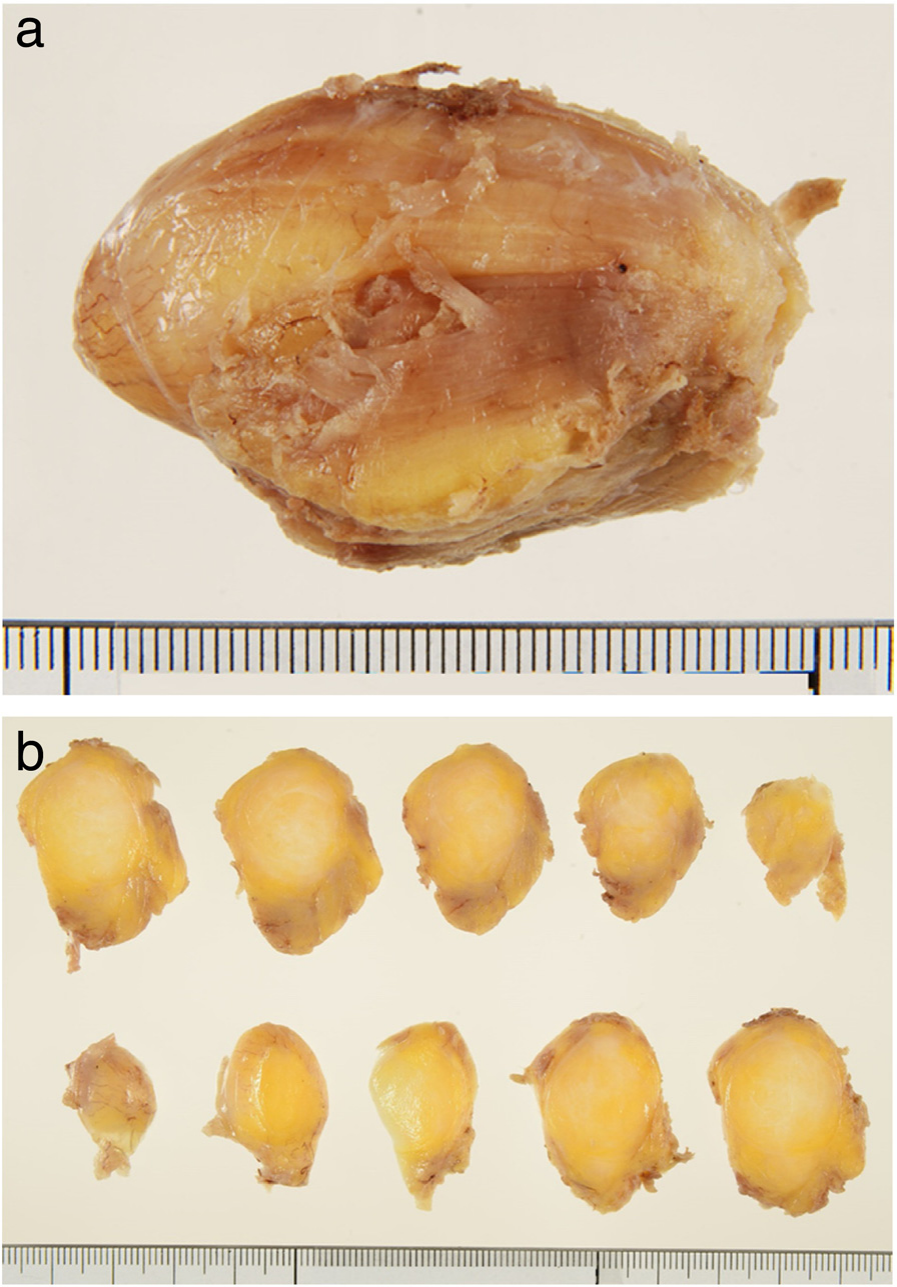
**Photographs of the tumor after formalin fixation. (a)** Formalin-fixed tumor. **(b)** The bisected surface of the tumor was whiter than that of a typical lipoma.

**Figure 5:**
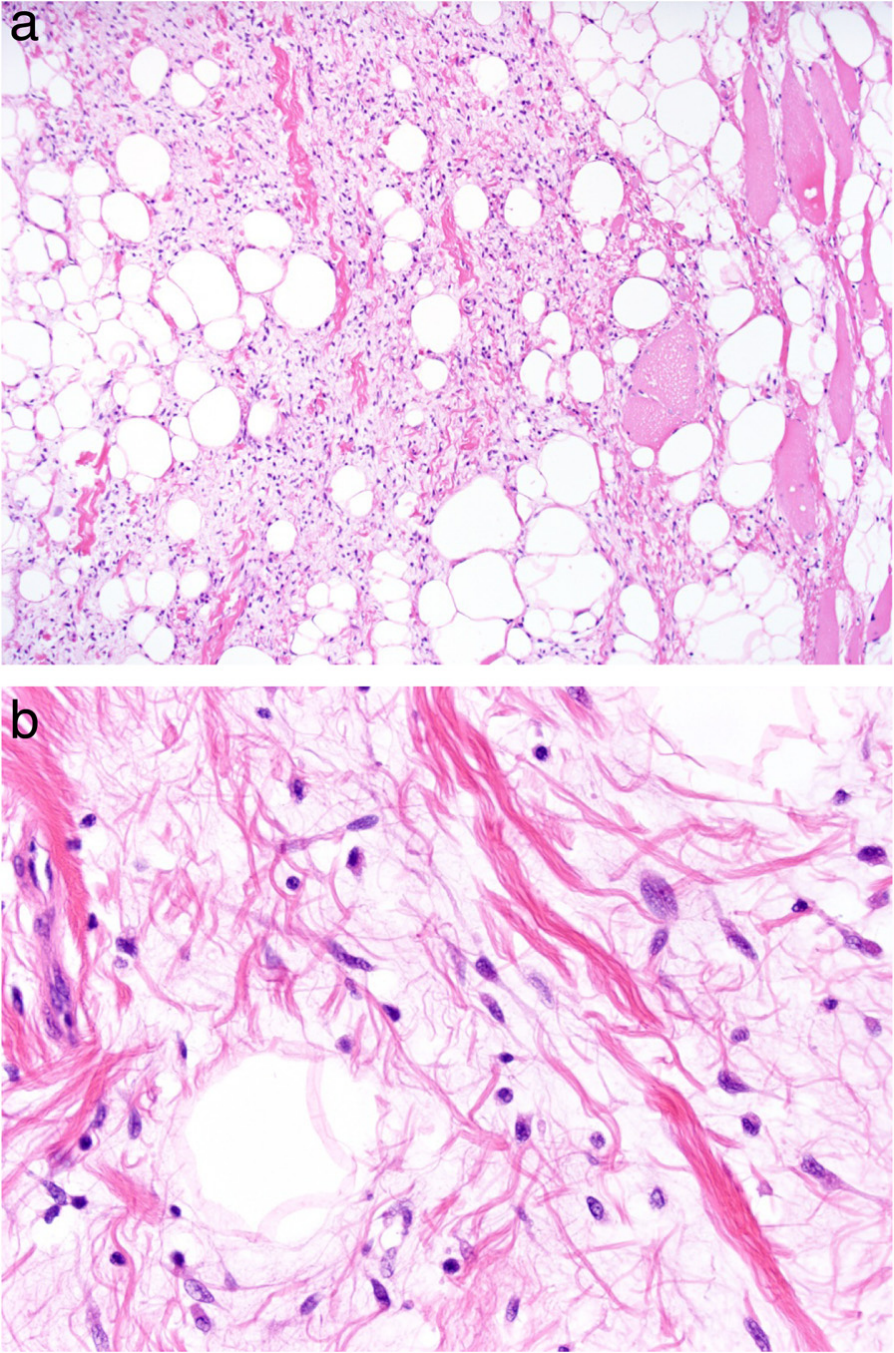
**Formalin-fixed, paraffin-embedded specimens were stained with hematoxylin and eosin for analysis.** The tumor consisted of spindle cells, collagen fibers and lipocytes. Original magnification, ×100 **(a)** and ×400 **(b)**.

On the basis of the pre-operative diagnosis of intramuscular lipoma, we removed the tumor with the patient under general anesthesia. During surgery, it was confirmed that the whole lesion was localized underneath the fascia and embedded within the deltoid. There was no adhesion to the surrounding tissues (Figure 2). The tumor was removed with surrounding musculature and fascia. The excised tumor was 5cm×4cm×3cm in size and had a yellowish color like that of a lipoma (Figure 3). However, it was slightly harder than a typical lipoma. The bisected surface was whiter than a typical lipoma (Figure 4a and b). To make a pathological diagnosis, formalin-fixed, paraffin-embedded specimens were stained with hematoxylin and eosin. The tumor consisted of spindle cells, collagen fibers and lipocytes (Figure 5a and b). The striated muscle fibers were infiltrated and entrapped by mature lipocytes in a diffuse manner. There were no lipoblasts or atypical cells. The results of immunohistochemical staining with MDM2, CDK4 and p16 were all negative. Therefore, we diagnosed intramuscular SCL of the left shoulder.

## Discussion

Lipomas are benign neoplasms of adult fat tissue, and they can be single or multiple and located superficially (subcutaneous) or deeper [5]. They are well-capsulated and slow-growing masses [5,6]. They can arise in any part of the human body [5-7]. There are several kinds of lipomas. Ordinary lipomas constitute 80% of lipomas; other types, such as angiolipoma, intermuscular and intramuscular lipoma, SCL, myolipoma, chondroid lipoma and pleomorphic lipoma make up the remaining 20% [5,6]. SCL is a relatively rare variant (1.5% of all adipocytic neoplasms) and is a histologically distinct lesion characterized by the replacement of mature fat by a mixture of mature adipocytes and undifferentiated spindle cells [1,5,8]. SCLs are typically well-circumscribed, benign, subcutaneous masses that arise in the neck, back and shoulders of middle-aged men [5]. In general, SCLs are not encountered in adolescents or children [5]. To our knowledge, there have been only five other cases of intramuscular SCL reported in the literature to date.

The age, sex and histopathological findings of our patient are all typical of SCL cases. The MRI scans of the lesion clearly show its location within the deltoid muscle. On MRI scans, the presence of nonlipogenic components in the lipogenic tumor may modify the radiological findings to some extent. When the proportion of nonlipogenic components such as spindle cells, collagen fibers and myxoid matrix is small, MRI findings of SCL are similar to those of conventional lipomas [8]. If the tumor contains many nonlipogenic components, the MRI findings may differ from those of typical lipomas. In our patient, the MRI findings differed from those of conventional lipomas, probably because the proportion of nonlipogenic components was large.

Histologically, SCLs are characterized by a mixture of lipocytes and fibroblast-like spindle cells [1]. These SCLs are uniform in size and have a single extended nucleus [1]. The most important part of the differential diagnosis is to distinguish SCLs from malignant lesions such as liposarcomas, because liposarcomas need more definitive treatment with wide excision with surrounding normal tissue. For differential diagnoses, including well-differentiated liposarcomas, particularly the sclerosing type, and myxoid liposarcomas is very important. Histologically, well-differentiated liposarcomas show lipocytes, spindle cells, collagen fibers and myxoid matrix. However, spindle cells in well-differentiated liposarcomas are more cellular and show nuclei with more pleomorphism than those seen in SCLs [1,5]. Therefore, we were able to exclude the diagnosis of well-differentiated liposarcoma and of other sarcomas.

The treatment method for SCL is marginal excision, together with the surrounding thin fibrous capsule [1]. As SCLs are benign tumors and there have been no reports of local recurrence, complete excision of the tumor results in a good prognosis.

## Conclusions

The present case is very interesting, as there have been few reported patients with a diagnosis of intramuscular SCL in a deltoid.

## Consent

Written informed consent was obtained from the patient for publication of this case report and any accompanying images. A copy of the written consent is available for review by the Editor-in-Chief of this journal.

## Abbreviations

MRI: Magnetic resonance imaging
SCL: Spindle cell lipoma
